# Multifunctional logic gates based on resonant transmission at atomic-plasmonic structure

**DOI:** 10.1038/s41598-022-15084-y

**Published:** 2022-06-24

**Authors:** M. Mosleh, S. M. Hamidi, M. Ranjbaran

**Affiliations:** 1 Magneto-Plasmonic Lab, Laser and Plasma Research Institute, Shahid Beheshti University, Tehran, Iran; 2 Department of Physics, Central Tehran Branch, Islamic Azad University, Tehran, Iran

**Keywords:** Optoelectronic devices and components, Optics and photonics, Optical data storage

## Abstract

Regarding the confinement of light at nanoscale dimensions in plasmonic structures, we try to show the impact of hot atomic vapor spectroscopy on a miniaturized scale. In such a combined structure, resonant coupling of the atom to plasmonic mode provides diverse ways to control the optical response of the system. We fabricate an atomic plasmonic cell based on Rubidium atomic vapor and gold plasmonic thin film onto the Kretschmann setup to introduce resonant coupling (EIT-like) of atom-plasmons as a tunable all-optical bandpass filter, switch, or logic gates. These all-optical devices such as NOR and XNOR logic gates are well done based on the filter by incidence angle of light, temperature as well as the external magnetic field. We believe the possibility of easy modulation of atomic susceptibility, not only through direct alteration on atoms but also through common methods available for modulation of plasmonic mode, has the potential to design and fabricate modern all-optical devices.

## Introduction

The idea of integration of atom as lab on a chip in miniaturized scale has attracted much interest over recent years to use them in appliances such as quantum computing, miniaturized quantum sensors, atomic clocks, etc.^1–5^. These studies cover effective control and optimal readout of atoms in micro or nano structures by in decreased volumes by optical near field of miniaturized structures^6–8^. Emergence of plasmonics with the possibility of confinement of light in conductive nano structures makes different intended and achievable photonic modes^9,10^. Photonic plasmonic modes have revealed many different properties, including complicated polarization state of near field as well as drastic enhancement of electromagnetic field near the surface. This makes surface plasmons (SP) a good candidate to all optically control and read out of atoms, thanks to large gradient potential of plasmonic modes in nano structures^11,12^. Touch of atoms by near field of SP’s gives rise to coupling of narrow discrete states of cooled and trapped or hot vapor of atoms with continuum state plasmonic resonator with different damping rates. The optical response of hybrid atomic-plasmonic system such as Fano resonance is strongly dependent on properties of both coupled oscillators^13,14^.

Fano resonance has been well studied in optical systems with multiple resonant modes and found more useful applications in sensing and switching fields and especially in atomic plasmonic area^15,16^. In this study, we investigate the tunability of Fano resonance of atomic-plasmonic system and introduce this coupled system as a paradigm for actively controllable bandpass line filter and atomic multichannel logic gates and switches.

Furthermore, in our atomic plasmonic systems, narrow optical atomic bandpass filters work based on induced transmission in atomic transition lines^17^. These narrow line filters are required in atmospheric LIDAR^18^, laser communication^19^, quantum optics^20^ or logic processing which is not possible without dynamic switching^21^. This means that having a filter with continuous and fast tunability with the possibility for integration on miniaturized solid-state devices will be especially important in the above-mentioned applications. Numerous types of physical mechanisms would be used to make narrow line atomic band pass filters. Well-known atomic line filters and switches are made based on magneto optical Faraday and Voigt atomic filters^22^ or electromagnetically induced transparency (EIT)^23,24^. We deduce physical similarities between EIT and atom-plasmon coupled resonant transmission (EIT-like) phenomena to propose a narrowband tunable bandpass filter and possibility to use a hybrid structure as a logic gate. Physical phenomena like four-wave mixing or slow light in atomic vapors employed to make ultra-narrow linewidth optical logic gates^25,26^. Following chip scale devices approach, integration of logic gates based on atomic vapor, to solid-state chips needs mediating a coupler of confined light to atomic media and confine treated signal back to chip, where will be lossy^27^. Many studies show best way to benefit atom-light interaction on an integrated chip, designs based on coupling of atoms with confined light in solid state^28^. One of promising approaches to design chip scale logic gates is harvesting interference of distinct confined plasmonic modes in metallic nanostructures to achieve “on”, “off” logic states, with benefit of solving mismatch between photonics and electronics^29^. There are some problems with plasmonic logic gates; one of them is increase instabilities in phase of interfering electromagnetic fields in extra low dimensions of structures, that causes low contrast ratio between produced logic states^30^. Another reported limitation of plasmonic logic gates is narrow bandwidth of these devices considering tens of nanometers in linewidth of plasmonic modes^31^. Here we show direct interaction of confined field with atoms has the possibility to carry logic operations regarding modulations over input states. Comparison of ultra-narrow linewidth absorption of Atoms (~ ${10}^{-3}$nm) with plasmonic modes (~ usually tens of nanometers) common phase instabilities in metallic nanostructure is ineffective in final coupled state. Also, possibility of integration of great number of separate atomic line channels in single plasmonic mode linewidth is an approach to solve narrow bandwidth of plasmonic-plasmonic logic gates keeping advantage of actively controllable plasmonic modes. In our designed setup, a change of plasmonic mode energy will control the transmittivity of filter and this technique would be extended rapidly over other atomic based devices considering principles of active plasmonics^32^. These principles could be a change of incidence angle, laser wavelength and temperature of metal thin film or dielectric function of surrounding media by external factors such as electric current, magnetic field, optical pulses and etc. or control on geometry of metallic nanostructures^33–35^. Also, as advantage of hybrid structure, disturbance on atoms such as strength and frequency of ac external magnetic field could be manipulated to resonantly control the transmitted light between on and off states.

## Material and methods

To study the reflection spectrum of coupled atomic-plasmonic modes, a combined structure of an Rb vapor cell and plasmonic substrate was fabricated. We designed a vapor cell with a coated prism as the optical window to guarantee direct touch between plasmonic near field and Rb atoms. For this purpose, glass prism was sputter-coated 35 nm of Au and to avoid direct contact and chemical reaction between Rb vapor and Au layer, 5 nm TiO_2_ was deposited on top of Au thin film. The combined atomic vapor cell was made by epoxy glue bonding of the finely polished ended borosilicate pipe on top of the prism, such that the coated part would be in the interior part of the cell. Then, hot vapor of Rb metal was injected into the cell and the end of the cell was terminated by a fire torch. To achieve the proper density of atomic vapor, the cell heated up to 85 °C. The heater was designed in a way to make a temperature gradient on the cell to prevent the solidification of Rb metal on the prism.

**Figure 1:**
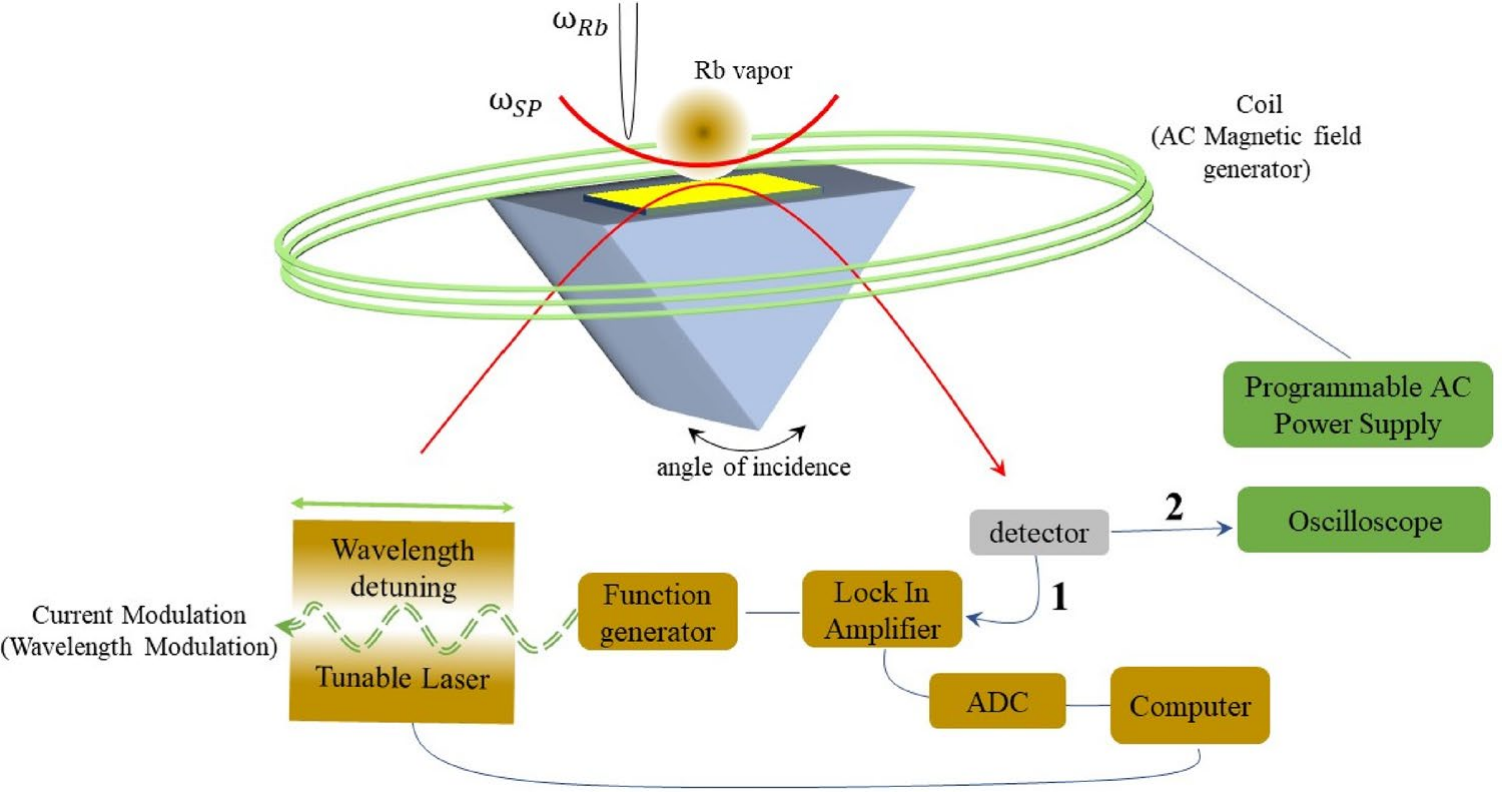
Part 1 (brown colored blocks) represent schematic diagram of the wavelength modulation spectroscopy used to measure reflectance from boundary of coated Au film and Rb vapor. Part 2 (green colored block) represent setup for modulation of magnetic field. Modulated electric current flows in represented coil and produces desired ac magnetic field applied to atom-SP boundary on prism.

To observe the resonant coupled transmission, we used the setup displayed in Fig. 1. Initially (part 1 as brown colored), we adjusted the incidence angle of distributed feedback (DFB) diode laser light onto the coated prism windows of Rb cell to excite SP wave. The DFB diode laser at the D_1_ line of Rb was frequency-modulated through driving current modulation. The laser light was reflected from the gold thin film-Rb atoms interface after passing through the Glen-Taylor prism. Then, the laser wavelength was finely scanned across the hyperfine structure to make a highly coherent plasmonic field interacting with the quantum states of rubidium atoms (As shown in Fig. 1).

The reflected light was captured by a photodetector connected to a lock-in amplifier. The lock-in amplifier was locked on the frequency of laser driving current and the intensity variation of the reflected light was recorded on a computer through a LabVIEW program while sweeping the laser wavelength. During part 1 of measurements and to make the hybrid atomic-plasmonic structure tunable, a rotation stage was employed to rotate the incidence angle corresponding to the SP resonance angle. We had sweep from −0.5 degree (before resonance) to 0 degree (SP resonance) and 0.5 degree (after resonance).

In part 2 (green colored devices) of our experiment, we tried to investigate the effect of ac magnetic field on coupled structure, a 500-turn coil with a diameter of 13 cm, and ac magnetic field of 5.4, 11.9, 29.5 mT were applied perpendicular to the SPP propagation direction.

## Results and discussion

**Figure 2:**
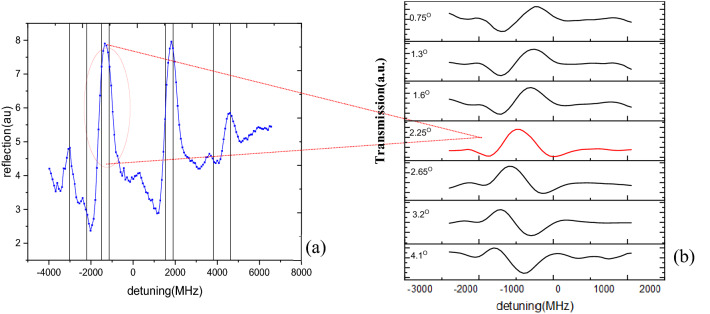
(**a**) Reflectance spectra of D1 hyperfine transitions in coupled atomic plasmonic structure. (**b**) angular modulation of selected transition line about 2.25 degree (SP resonance induced angle of incidence), for selected hyperfine transition line of rubidium metal marked by red ellipse.

Figure 2 reveals the reflection spectrum of our hybrid structure while SP’s touched the Rb atoms with modal energy equal to Rb’s hyperfine transitions energy. Based on the large damping rate of plasmons, we neglect changes of SP’s energy in narrow range of Rb D1 hyperfine transitions. The results clearly show transmission of incoming light in wavelength detuning equal to any atomic hyperfine transition. The fact is, all incoming photons after passing the prism windows couple to plasmonic mode and get damped in different channels especially by ohmic damping in metal film. The transmitted photons of our structure scape plasmonic damping by resonant coupling to hyperfine transitions of atoms. The physical behavior of resonance in coupled atom-SP system proves resonant transmission (EIT-like) regime of our proposed hybrid structure acts on EIT based filters platform for a band-pass filter with FWHM of line, equal to atomic transitions. Also, we believe any physical change leading to sensible decrease in line width of atomic transmissions, such as selective reflection, or plasmonic saturation absorption would be utilized alongside SP’s to achieve a narrower band-pass filters, while taking advantage of coupling paradigm.

To analyze tunability of the combined behavior of coupled atom-SPP transmittivity, we performed spectroscopic measurements in two steps; first under a shift of SP mode energy and second by modulating quantum state of Rb atoms by applying an ac external magnetic field. To change the energy of SP’s formed at metal–dielectric interface in Kretschmann configuration, the easiest way is altering the angle of incidence of laser light (*Supplementary information S1-S2*). Figure 2a represents measurements done in an all fixed experimental conditions where only angles of incidence of photons changed around SP resonance angle. Figure 1b clearly shows a sharp change of results of coupled system by changes in incidence angle for wavelength equal to 5^2^S_1/2_–5^2^P_1/2_ atomic transitions of ^85^Rb isotope. While a change of angle of incidence of laser light alters energy of SP, the coupling regime turns from the Fano to EIT-like and vice versa. We would conclude that behavior of the filter changes from a band pass filter to an absorbing line filter. It happens because a major change in incidence angle causes overthrow of plasmonic mode and optical near-field turns from SP to evanescent field of dielectric prism, so commonly atomic absorbance take place as is obvious in the last row of data.

SP modulating mechanisms would be used to tune atom plasmon coupling to exploit a hybrid device like the proposed filter. This includes variety of engineered plasmonic structures available in published papers^36^. In addition to the effect of change in angle as presented, we examined the effect of increase in temperature of measurement examination in possible ways on single Au thin film. We compare the result of measurement in 85 and 105 degrees Celsius. Harsh increase in temperature actually eliminates condition of coupling of atom-SP’s due to increases in Ohmic damping of Au film. In response, the rate of energy damping of photons (polaritons) at SP mode overtakes the rate of energy exchange between atom and SP. With elimination of associated optical susceptibility of coupled structure, optical response of atoms to evanescent wave of total reflection from prism became dominant and resonant transmission behavior disappeared due to breakdown of coupling condition. (*Supplementary information, page S-3*).

As explained in the experimental part, incidence angle to atom-plasmonic cell clearly shows resonant transmission of photons from coupled structure by all possible Rb atom hyperfine transitions. Transmittivity is directly proportional to amplitude of both resonances specially, atomic hyperfine transition probability. Considering multi lines of hyperfine absorptions, with different levels of transmittivity caused by Rb atoms, we propose logic behavior of atom-SP coupling. To show the effects of impressive parameters, we set the incidence angle of laser light to prism at, −0.5, −0.27, −0.17 degree (off SP resonance), 0 degree (SP resonance) and 0.08, 0.17, and 0.29 degree (off SP resonance) and recorded the reflected laser light intensity in swept wavelengths (detuning marked as x axis of the results). To define normalized incidence angle, we consider SP resonance angle, 2.25 degree, as the reference ones and for each incidence angle we use $\frac{\theta -2.25}{\theta +2.25}$.

**Figure 3:**
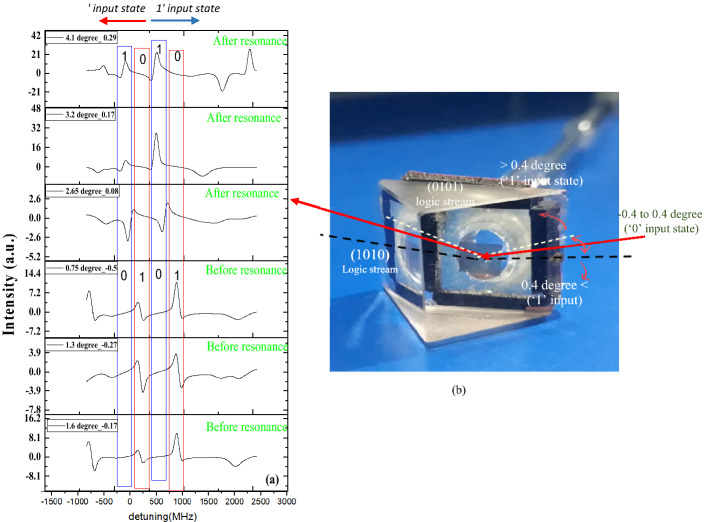
(**a**) reflectance signal from the interface of SP- Rb atomic vapor by wavelength detuning of laser light, under shifts of incidence angle of laser light. (**b**) Produced atomic-plasmonic cell with schematic arrows to represent the possible range of incidence angle, considering input logic states. The red solid arrows indicate the SP resonance incidence angle while the dotted white line shows −0.4 degree before resonance (0101 logic stream) and black dotted line shows 0.4 degree after resonance (1010 logic stream). The range of incidence angles which are considered as 0 and 1 logic inputs are depicted as red curved arrows. All amounts of incoming angles are measured from angle of SP resonance.

The results show EIT-like transmission at SP resonance angle (Fig. 2a) and asymmetric Fano resonance at off resonance angles in wavelength of any single hyperfine transitions line. Figure 3a depicts the reflectance normalized to the EIT-like reflectance of laser light over hyperfine transitions at any incident angle. To investigate possibility of multichannel switching, we consider any single D_1_ hyperfine absorbance lines of Rb atom as a distinct gate. In Fig. 3a, four blue and red colored wavelength channels are marked. We would assign 0 or 1 logic values depending on reflectance intensity value in each of channels. Comparison of our results in Fig. 3b shows ‘1010’ logic stream for after resonance and ‘0101’ logic value before resonance angles. The change of symmetry of the logic state stream is caused by the sweep of the incidence angle around the SP resonance angle. The energy of SP mode in resonance angle equals the atomic transition energy, increase or decrease in angle of incidence causes an increase or decrease in SP energy related to the energy of atomic transitions. The sign of symmetry of the Fano line profile in each channel directly depends on the sign of energy difference of SP mode from Rb transitions. The effect of imposing change on incidence angle is not only on the sign of symmetry, the value of asymmetry of Fano line shape is directly proportional to the difference between coupled modes energies^16^. This change drifts us to introduce atom-SP hybrid structure as an optical switch which would alter the logic state of channels only by a change on incidence angle about SP resonance angle.

**Figure 4:**
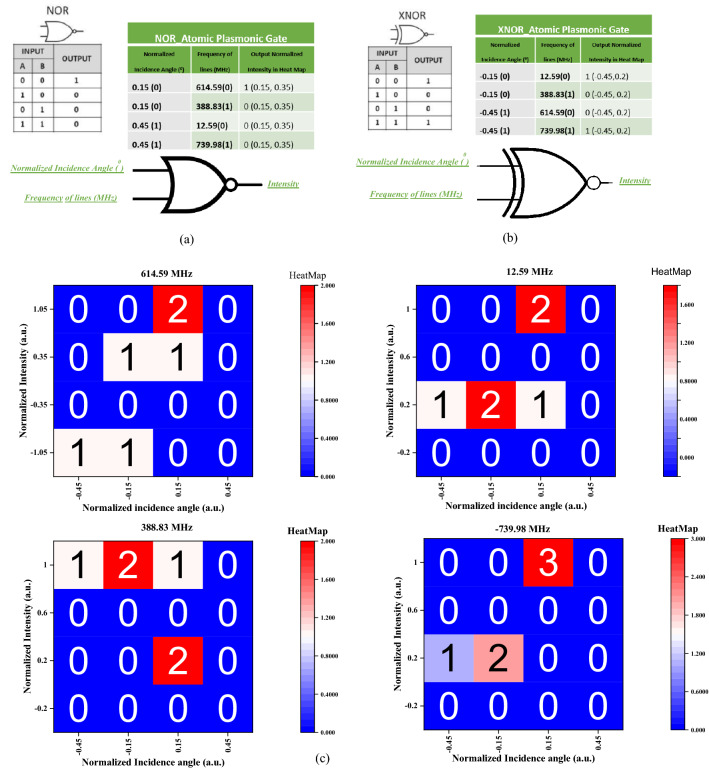
operation of proposed (**a**) NOR and (**b**) XNOR logic gates and (**c**) Heat Maps of logic state based on transmitted intensity at four selected detuning channels of system's functionality.

Here to investigate our proposed logic gate performance, we define system inputs as incidence angles and the wavelength detuning. Figure 4 reveals a result of Heat Map, in which, normalized angle and wavelength are the first and second inputs, and reflectance intensity is output channel. We supposed the incidence angles in the range of [−0.4, 0.4] degrees as 0 logic input and all other angles off this range as 1 logic input. In addition, the frequency detuning lines ν < 300 MHz, are supposed to be 0 and distinct lines as 1 logic states. In this way, we would construct four 00, 01, 10, and 11 logic input states, as is done in Fig. 4.

The results show that our hybrid structure would act as XNOR or NOR logic operator only by switch on the frequency of incoming light and apply a change on SP mode energy.Here, the change of incidence angle of the incoming light is used to control SP modal energy, surly the rate of mechanically applied change on incidence angle is totally lower than the ideal atom- SP based switching system. In our proposed structure damping rate of Rb hyperfine transitions (couple of GHz) and lifetime of an exited SP mode (femtosecond) will be the ultimate limiting parameters on the ON–OFF switching rate of an ideal atom-SP based logic gate^29,37^.

As is expected from coupled structures behavior of both sides is effective in behavior of hybrid structure. Thus far, we have shown the effect of changes on SP mode in coupled susceptibility of atom- SP coupled media. Any disturbance in quantum states of Rb atom alters the operation of the coupled behavior too.

Applying a magnetic field breaks up the degeneracy of hyperfine levels and split them into Zeeman sublevels according to their quantum number. Amount of applied magnetic field directly controls amount of splitting and shifts transition energy between quantum state. So, the absorption spectrum of the D1 line of Rb vapor would be changed associated with the strength and direction of the applied magnetic field. Consequently, when the wavelength and incidence angle of input light is fixed, the output light intensity could be controlled by applying a magnetic field. In fact, the magnetic field strength can play the role of the control signal in the proposed structure.

**Figure 5:**
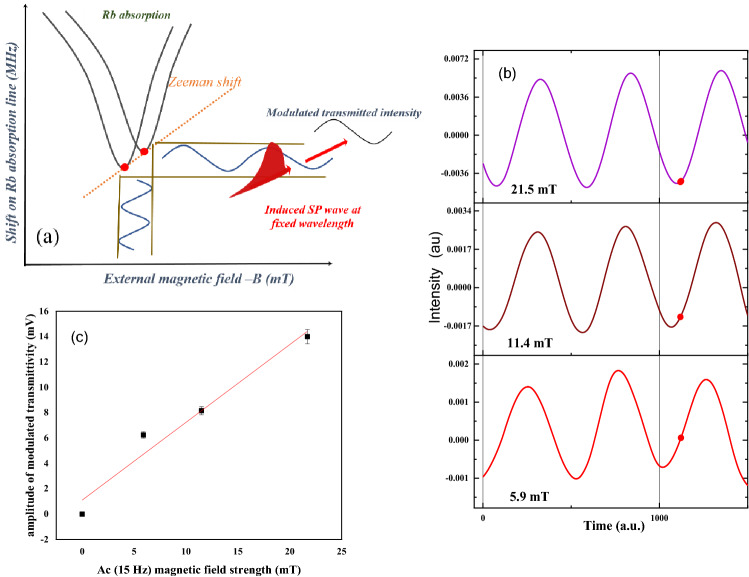
(**a**) Schematic diagram of transmitted light modulation from SP- atom coupled system, as applied modulated B field alters absorption line of atom over Zeeman shift. (**b**) Transmittivity of Rb atom -SP system at three different strength of applied ac magnetic field. Red colored point shows the phase change of modulated transmittivity at different strengths of applied field, B. (**c**) Amplitude of modulated transmittivity versus strength of magnetic field. In the domain of small changes of magnetic field strength, the sensitivity of transmittivity to ac B field strength is linear.

In our case, a linearly polarized light was incident on the gold coated prism and SP wave optically pumped the Zeeman sublevels, while the magnetic field was applied perpendicularly to the propagation direction of the SP’s. Implementing frequency modulation technique, Rb hyperfine transitions for Rb D_1_ line were completely resolved. However, due to the Doppler broadening, at low strength of the magnetic field (lower than ~ 10 mT for Rb), the allowed transitions between large numbers of magnetic sublevels could not be spectrally distinguished. So, instead of applying an external DC magnetic field, we studied the transmittivity of the coupled atomic-plasmonic system, under the application of ac magnetic field, at a fixed wavelength of incoming light. A modulated signal was obtained by recording the light intensity while the laser wavelength was retained at one of the hyperfine transitions and the ac magnetic field was applied on Rb vapor (Fig. 5a). We recorded changes for three different 15 Hz ac magnetic fields at amplitudes of 5.9, 11.4, and 21.5 mT. The results showed that modulation depth of transmitted laser light was proportional to the strength of the applied magnetic field. Although in all cases the transmitted light was modulated by the same frequency, a small phase shift was observed in the results (Fig. 5b).

Our results also show the possibility of the change in transmittivity and phase of our proposed gate by a variation in the amplitude of the applied ac magnetic field. As displayed in Fig. 5b, red dot shows the phase changes of transmittivity in a coupled atomic plasmonic structure and offers the possibility of phase modulation of logic by variation of the strength of the magnetic field. It is worth mentioning that the Zeeman splitting-based-shift of an absorption line is not linearly related to the strength of the applied magnetic field. So, we used weak strength of the magnetic field to perform light modulation in a linear regime. Figure 5c shows relation between the amplitude of modulated laser light in transmission from the coupled system and the strength of applied magnetic field. Extrapolation of this figure indicated the sensitivity of amplitude modulation of transmitted light to magnetic field strength is in the order of 0.62 V/T.

## Conclusion

Considering the impact of integration of atomic-photonic operations on solid state devices we tried to show advantage of coupled atomic-plasmonic structures based on possible control parameters such as modal energy of SP waves, temperature of interaction medium, applied external magnetic field and the wavelength of incoming light. Our experimental results show tunability of a combined atomic-plasmonic susceptibility is an in-hand candidate to define switchable atomic line filters. Coupled narrow bandpass line filters would be easily turned on and off by change of plasmonic mode energy. Also, we show our proposed filters take advantage of modulation of transmittivity by applying an external magnetic field. In completion of studies on bandpass filters according to this approach, we tried to show the logic relation between control parameters in a combined structure based on mentioned degrees of freedom. We believe this paradigm could be used in the design of the scalable logic gate, such as all optically plasmonic logic gates to achieve wider bandwidth and increased external controllability while eliminating disorders. At the end, we believe the mix of fascinating applications of atomic hot vapor spectroscopy with widespread human knowledge on plasmonics would be strongly beneficial.

## Supplementary Information

Supplementary Information.

## Data Availability

Data underlying the results presented in this paper are not publicly available at this time but may be obtained from the authors upon reasonable request.
