# Supplementary material for: Multifunctional logic gates based on resonant transmission at atomic-plasmonic structure
Source: Sci Rep. 2022 Jun 24;12:10734. doi: 10.1038/s41598-022-15084-y (PMC9232648; doi:10.1038/s41598-022-15084-y)
Supplement: Supplementary file 1 — Supplementary Information. [file 41598_2022_15084_MOESM1_ESM.docx]

**Supplementary information:**

**Multifunctional Logic Gates based on Resonant Transmission at Atomic-Plasmonic structure**

M. Mosleh^1^, S. M. Hamidi^1,*^, M. Ranjbaran^2^

^1^ Magneto-plasmonic Lab, Laser and Plasma Research Institute, Shahid Beheshti University, Tehran, Iran.

^2^Department of Physics, Central Tehran Branch, Islamic Azad University, Tehran, Iran.

*m_hamidi@sbu.ac.ir

Change in SP’s energy by deviation on angle of incidence:

Coherent excitations of surface plasmon polariton waves start to exist on boundary of metal thin film / dielectric medium, if phase matching condition between incoming light and potential bounded photonic mode (plasmonic mode) fulfills. To satisfy phase matching condition we exploited Kretschmann-Raether configuration as is shown in Fig 1. Of the main manuscript. Excitation of Surface plasmons unveils itself as a minimum on reflection versus incidence angle. To show the effect of a small change in angle of incidence about critical angle, on energy of SP resonance we investigate classical equation of motion of damped oscillator,

$$m\frac{d^{2}x}{dt^{2}}+\gamma\frac{dx}{dt}+kx=0$$

This equation has an answer in the form of,

$$x\left( t \right)=Ae^{i\omega t}e^{-\alpha t}=Ae^{i\Omega t}$$

$$\Omega=\omega+i\alpha$$

$\Omega$ is complex frequency of resonance. If the above answer inserted in equation of motion after mathematical calculations, the frequency of resonance of damped oscillator (SP) will be in the form,

$$\omega_{res}={({\omega_{0}}^{2}-\alpha^{2})}^{\frac{1}{2}}$$

where,

$\omega_{0}=\sqrt{\frac{k}{m}}$ , $\alpha=\frac{\gamma}{2m}$

Calculation represents that for a damped oscillator frequency of resonance is slightly different from natural resonance frequency of undamped oscillator ($\omega_{0}$) as is obvious from expression for $\omega_{res}$. This means dynamic change of damping of oscillator will cause modulation on frequency of resonance. It’s worth to mention the same route is repeatable for damped driven oscillator and the effect of damping will be appeared as a change in phase of response oscillation.

SP’s are the form of resonance with two different type of damping known as leakage damping ($\gamma_{L}$), because of leakage of energy from SP modes back to prism, and internal damping of metal ($\gamma_{int}$), rising from Ohmic damping of metal plus interband transitions. Change of angle of prism related to impinging photons directly causes change on leakage damping of SP’s, while the internal damping stays fix. Its worth to mention minimum of reflection take place at critical angle when amount of $\gamma_{L}$ equals $\gamma_{int}$ and FWHM of SP resonance is 2$\gamma_{int}.$

Until here we tried to show a change in angle of incidence of light on Kretschmann configuration effectively changes the net damping of SP resonance mode. According to above mentioned calculation, this is equivalent to small change of resonance frequency. This is main reason to observe Fano- EIT(like)- Fano interference in our hybrid system only by changes of angle of incidence (Figures 1 and 2S)

Fano

Fano

EIT-Like

2 $\gamma_{int}$

Figure 1S. Simulated absorption of SP at 35nm of Au layer on prism. The red indicator under diagram shows experimentally observed physical phenomena related to changes of angle of incidence around critical angle.

Figure 2S: Reflectance spectra at three different incidence angle before, at and after resonance.

Effect of harsh increase in temperature at coupling process:

The effect of big increase in temperature during our experiments manifests itself as a big change of recorded data into simple absorption of rubidium atoms. We believe that main reason for this happening is breakdown in coupling condition caused by increase in internal damping of metal thin film, specially ohmic damping that causes rate of SP energy decay advantage rate of energy transfer between SP and atoms. Diagram below shows absorption of Rb atoms by evanescent field of total internal reflection of light from prism/Au boundary. Results clearly shows conversion of transmission of coupled structure to simply evanescent wave absorption. (it’s worth to mention existence of Au layer between evanescent field and Rb atoms mainly decrease signal to noise ratio)

Figure 3S. Reflection spectra of atomic plasmonic cell before and after enhance in temperature.

**References:**

[1] Nettel, S. *Wave Physics: Oscillations, Solutions, Chaos*. Springer Science & Business Media (2003).

[2] Maier, S. A. *Plasmonics: fundamentals and applications* New York: springer. (2007).
